# Microbial signatures predictive of short-term prognosis in severe pneumonia

**DOI:** 10.3389/fcimb.2024.1397717

**Published:** 2024-08-02

**Authors:** Shen-Shen Huang, Jia-Yong Qiu, Shuang-Ping Li, Ya-Qing Ma, Jun He, Li-Na Han, Long-Long Jiao, Chong Xu, Yi-Min Mao, Yong-Mei Zhang

**Affiliations:** ^1^ Department of Respiratory and Critical Care Medicine, The First Affiliated Hospital, and College of Clinical Medicine of Henan University of Science and Technology, Luoyang, China; ^2^ Department of Respiratory and Critical Care Medicine, The Second Hospital of Hebei Medical University, Shijiazhuang, China

**Keywords:** severe pneumonia, lung microbiota, short-term prognosis, microbial diversity, pathogenic bacteria, machine learning

## Abstract

**Objective:**

This retrospective cohort study aimed to investigate the composition and diversity of lung microbiota in patients with severe pneumonia and explore its association with short-term prognosis.

**Methods:**

A total of 301 patients diagnosed with severe pneumonia underwent bronchoalveolar lavage fluid metagenomic next-generation sequencing (mNGS) testing from February 2022 to January 2024. After applying exclusion criteria, 236 patients were included in the study. Baseline demographic and clinical characteristics were compared between survival and non-survival groups. Microbial composition and diversity were analyzed using alpha and beta diversity metrics. Additionally, LEfSe analysis and machine learning methods were employed to identify key pathogenic microorganism associated with short-term mortality. Microbial interaction modes were assessed through network co-occurrence analysis.

**Results:**

The overall 28-day mortality rate was 37.7% in severe pneumonia. Non-survival patients had a higher prevalence of hypertension and exhibited higher APACHE II and SOFA scores, higher procalcitonin (PCT), and shorter hospitalization duration. Microbial α and β diversity analysis showed no significant differences between the two groups. However, distinct species diversity patterns were observed, with the non-survival group showing a higher abundance of Acinetobacter baumannii, Klebsiella pneumoniae, and Enterococcus faecium, while the survival group had a higher prevalence of Corynebacterium striatum and Enterobacter. LEfSe analysis identified 29 distinct terms, with 10 potential markers in the non-survival group, including Pseudomonas sp. and Enterococcus durans. Machine learning models selected 16 key pathogenic bacteria, such as Klebsiella pneumoniae, significantly contributing to predicting short-term mortality. Network co-occurrence analysis revealed greater complexity in the non-survival group compared to the survival group, with differences in central genera.

**Conclusion:**

Our study highlights the potential significance of lung microbiota composition in predicting short-term prognosis in severe pneumonia patients. Differences in microbial diversity and composition, along with distinct microbial interaction modes, may contribute to variations in short-term outcomes. Further research is warranted to elucidate the clinical implications and underlying mechanisms of these findings.

## 1 Introduction

Pneumonia represents a significant global health burden, contributing substantially to morbidity and mortality worldwide (Ventola, 2015; Liu et al., 2016; Diseases and Injuries, 2020). Severe pneumonia-related mortality is a significant cause of in-hospital death among patients. It is estimated that approximately 100,000 pneumonia patients require admission to the intensive care unit (ICU) for mechanical ventilation (MV) annually (Spindler and Ortqvist, 2006; Mandell et al., 2007). Among severe pneumonia patients admitted to the ICU, the risk of mortality is highest, with approximately 20-50% of ICU pneumonia patients succumbing to the illness (Rodriguez et al., 2009; Valles et al., 2014; Walden et al., 2014; Lee et al., 2020). Timely and accurate determination of the etiology of pneumonia is imperative for initiating targeted therapeutic interventions effectively. However, conventional microbiological tests currently used often exhibit limitations in terms of sensitivity, speed, and the breadth of detectable pathogens (Jain et al., 2015). For instance, even with optimal clinical diagnostics, only 38% of adults with community-acquired pneumonia have a contributory pathogen detected, primarily due to the limitations of culture-based methods and the restricted spectrum of microbes detectable by serologic and polymerase chain reaction assays (Jain et al., 2015; Song et al., 2016). In the absence of a definitive microbiologic diagnosis, clinicians may resort to empiric treatments, such as corticosteroids, potentially exacerbating occult infections (Cilloniz et al., 2021). Moreover, concerns about falsely negative results often lead to the continued use of empiric antibiotics, contributing to antibiotic resistance and increasing the risk of secondary infections (Antimicrobial Resistance Collaborators, 2022). Despite early antimicrobial treatment and support measures, mortality due to severe pneumonia is still very high and new approaches in respiratory therapy are being sought to try to improve their outcomes (Prina et al., 2015).

Recent advancements in genome sequencing offer promise in addressing these diagnostic challenges by enabling culture-independent assessment of microbial genomes from minute clinical samples (Chiu and Miller, 2019). Meta-genomic next-generation sequencing (mNGS) has emerged as a valuable tool for the rapid and actionable diagnosis of complicated infections, including pulmonary infectious (Li et al., 2020; Shi et al., 2020) or distinguished noninfectious diseases (Peng et al., 2021). Several studies have demonstrated the utility of mNGS in improving the diagnosis of pulmonary infectious by identifying a broader range of pathogens than conventional methods, including bacteria, viruses, fungi, and atypical organisms (Chen et al., 2021; Jin et al., 2022; Meng et al., 2023). For example, a recent multicenter study reported that mNGS detected pathogens in approximately 80% of cases where conventional methods failed to yield a diagnosis, highlighting its superior sensitivity compared to traditional approaches (Xie et al., 2021). Moreover, mNGS has shown promise in guiding antimicrobial therapy decisions by providing rapid and comprehensive pathogen identification, thereby facilitating targeted treatment strategies and potentially reducing the unnecessary use of broad-spectrum antibiotics (Xu et al., 2023).

Beyond its role in pathogen detection, mNGS offers valuable insights into the lung microbiome, which plays a critical role in maintaining respiratory health and modulating immune responses (Budden et al., 2019; Natalini et al., 2023). Emerging evidence suggests that dysbiosis of the lung microbiome, characterized by alterations in microbial composition and diversity, may contribute to the pathogenesis of pneumonia and influence disease outcomes (Montassier et al., 2023). Furthermore, changes in the lung microbiome have been observed in various disease states, including chronic obstructive pulmonary disease (COPD) (Wang et al., 2016), asthma (Hufnagl et al., 2020), and cystic fibrosis (Cuthbertson et al., 2020), underscoring the importance of understanding microbial dynamics in respiratory health and disease.

Machine learning (ML) techniques have also been increasingly applied to microbiome data to predict disease outcomes and guide clinical decisions. ML algorithms can integrate complex datasets, identifying key molecular or microbiome signatures, improving diagnostic accuracy and unfavorable clinical outcomes (Li et al., 2022). These predictive models can enhance the speed and precision of pneumonia diagnosis, enabling personalized therapeutic interventions (Montassier et al., 2023).

Given the potential of mNGS to provide comprehensive insights into both pathogen detection and lung microbiome composition, there is growing interest in exploring its role in predicting disease outcomes and guiding personalized therapeutic interventions. However, the relationship between microbial dysbiosis and clinical outcomes in severe pneumonia remains poorly understood. Therefore, the aim of this study is to investigate the association between changes in the lung microbiome and 28-day mortality in patients with severe pneumonia using mNGS technology. By analyzing microbial composition data obtained through mNGS alongside clinical parameters, we seek to identify microbial biomarkers associated with disease severity and patient outcomes. Insights gained from this research could contribute to the development of personalized management strategies and enhance the prognostic accuracy of severe pneumonia, ultimately leading to improved patient outcomes.

## 2 Materials and methods

### 2.1 Study design

This retrospective study enrolled critically ill pneumonia patients admitted to the Respiratory Intensive Care Unit (RICU) of the First Affiliated Hospital of Henan University of Science and Technology from February 1, 2022, to January 30, 2024. The Inclusion Criteria included; (1) Patients were included if they met the diagnostic criteria for severe community-acquired pneumonia (CAP) according to the American Thoracic Society and Infectious Diseases Society of America(ATS/IDSA) guidelines (Metlay et al., 2019) for the diagnosis and treatment of adult community-acquired pneumonia; (2) The enrolled patients had bronchoalveolar lavage fluid (BALF) samples collected for mNGS testing; (3) Patients with complete clinical data. While, the Exclusion Criteria: patients under the age of 18, those with repeated tests of mNGS (only the first result was chosen), those with incomplete clinical data or medical history, samples that failed to pass the quality control of mNGS, and patients who died due to treatment abandonment were excluded. All patients diagnosed with severe CAP receive empirical antimicrobial therapy according to the guidelines upon admission. Furthermore, comprehensive assessments including blood routine tests, procalcitonin (PCT), C-reactive protein (CRP) levels, microbiological analysis, microculture, and chest imaging examinations are promptly conducted. Treatment strategies are adjusted based on the results microculture and mNGS results.

**Figure 1:**
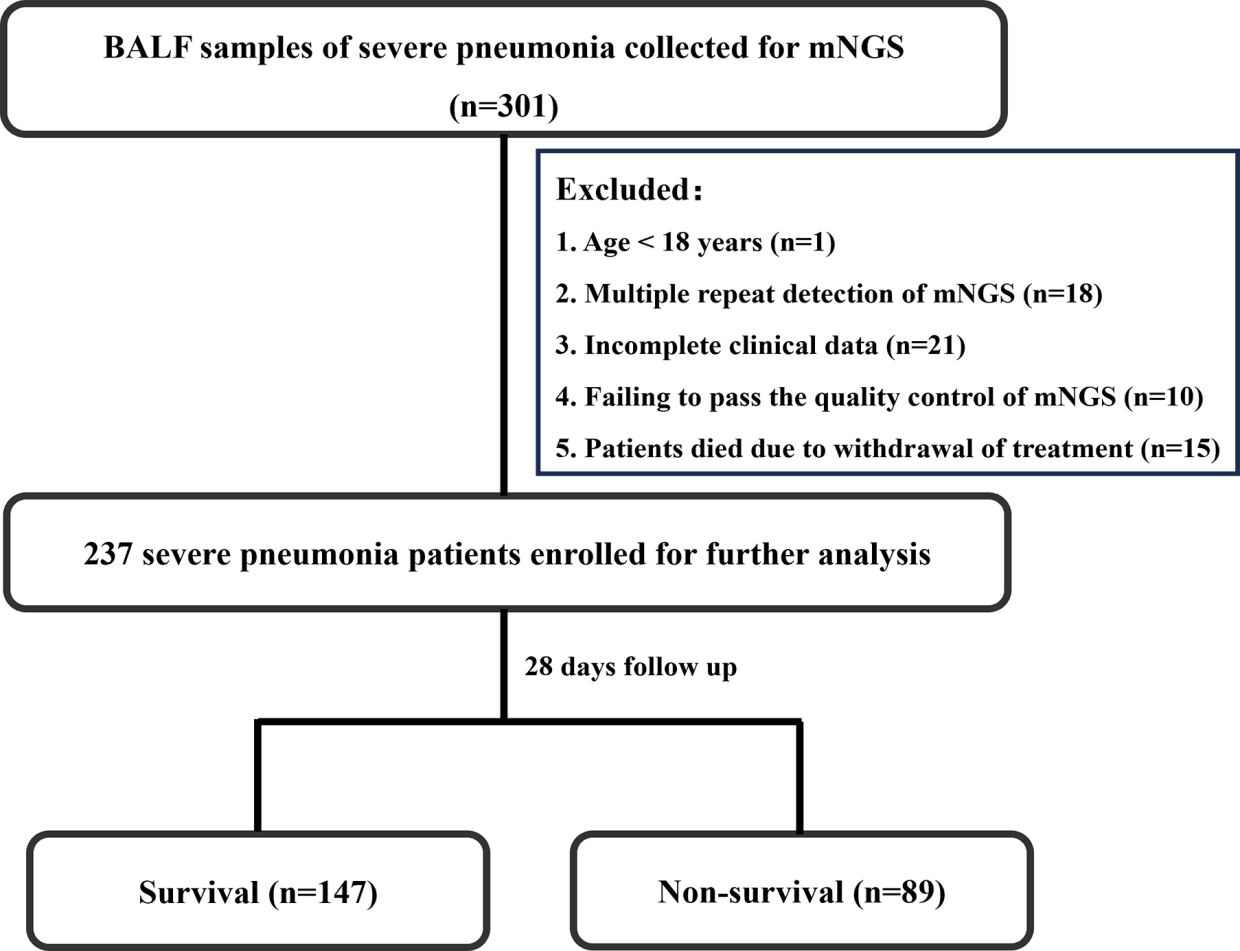
Flowchart.

Bashed on the mortality of 28 days after admission to hospital, the enrolled patients were divided into two groups, that is survival group and non-survival group. The flowchart of patient enrollment is illustrated in **Figure 1**.

### 2.2 Clinical data and outcomes collection

Baseline clinical characteristics and outcomes were collected retrospectively for each enrolled patient upon admission. This included gender, age, acute physiology and chronic health evaluation II (APACHE II) score, sequential organ failure assessment (SOFA) score, presence of comorbidities (such as hypertension, diabetes mellitus, coronary artery disease, chronic obstructive pulmonary disease[COPD], stroke, cancer, etc.), laboratory parameters (including white blood cell count, neutrophil count, platelet count, CRP, PCT, etc.), length of hospital stay, duration of ICU stay, duration of MV, and 28-day survival status.

### 2.3 Sample collection

BALF samples were obtained by respiratory physicians via bronchoscopy guided by the pulmonary imaging findings of the patients within two days after administration. A minimum of 2-3 mL of BALF was collected and stored in sterile containers for subsequent mNGS analysis.

### 2.4 DNA extraction and mNGS sequencing procedure

BALF specimens (1.2 mL) were mixed with 12 μL of BALF in 2 mL centrifuge tubes and subjected to cell lysis using a sample oscillation disruptor (BSP-100, Hangzhou Jieyi Biotechnology Co., Ltd., China). Subsequently, the samples were centrifuged at 12000 rpm for 3 minutes (LX-200, Haimen Qilin Bell Instrument Co., Ltd., China), and 400 μL of the supernatant was transferred to a genomic DNA extraction or purification kit (MD013, Hangzhou Jieyi Biotechnology Co., Ltd., China), with additional corresponding reagents added as required. The samples were then processed in an automated nucleic acid detection reaction system (NGSmasterTM library preparation, MAR002, Hangzhou Jieyi Biotechnology Co., Ltd., China) for nucleic acid extraction (RNA reverse transcription), enzymatic digestion, end repair, end adenylation, adapter ligation, and library construction. Quantitative analysis and pooling of the established DNA libraries were performed using real-time PCR (KAPA method). The quantified DNA libraries were subjected to high-throughput sequencing on the Illumina Nextseq system (Nextseq 550, Illumina, Inc., USA), generating approximately 20 million 50-base pair single-end reads for each library.

### 2.5 Bioinformatic process

The sequencing data underwent initial demultiplexing to isolate the sequence reads of each sample in fastq format. Subsequently, high-quality sequencing data was obtained by filtering out short reads (<35 bp), low-quality reads, and reads with low complexity. The sequence reads of each sample were then aligned to the human reference genome (GRCh38.p13) to eliminate human sequences by using bowtie2 (Langmead et al., 2019). Microbial species identification was conducted based on the analysis of clean reads using Kraken2 (Wood et al., 2019). Alpha diversity metrics including Shannon’s index and Chao1, along with beta diversity metrics, principal coordinate analysis (PCoA), were calculated using R (version 4.3.0). Furthermore, linear discriminant analysis effect size (LEfSe) was employed to identify the features most likely responsible for the differences between the groups (Segata et al., 2011).

Based on species identified by kraken2 alignment, we employed five machine learning methods to construct predictive models (Jaafari et al., 2022; Yu et al., 2022): random forest (RF), support vector machine (SVM), generalized linear model (GLM), multivariate adaptive regression splines (MARS), and regularized random forest (RRF). We then utilized the “DALEX” package in R to analyze the five models and their residual distributions, and plotted receiver operating characteristic (ROC) curves to determine the best-performing model. Subsequently, the selected best model was used to identify key microbial species associated with 28-day mortality in severe pneumonia cases.

Furthermore, we employed the best model in R to identify key bacteria that differentiate between the two groups of samples. A microbial network was also constructed by retaining edges with correlation coefficients (R) ranging between -0.8 and 0.8, with a significance threshold of P < 0.05. The analysis was conducted using the igraph package in R and visualized by Gephi (Bastian et al., 2009) accordingly.

### 2.6 Statistical analysis

All statistical analyses were conducted using SPSS version 23.0. Descriptive statistics were used to summarize the data, with categorical variables presented as numbers (percentages) and continuous variables expressed as means ± standard deviations or as medians (interquartile ranges). Group comparisons for categorical variables were performed using the chi-square test or Fisher’s exact test, while continuous variables were analyzed using Student’s t-test for normally distributed data and the Wilcoxon rank-sum test for non-normally distributed data. Variables with a univariate analysis A *P*-value < 0.05 was considered statistically significant.

## 3 Results

### 3.1 Baseline characteristics

This study is a retrospective cohort study that consecutively enrolled 301 patients diagnosed with severe pneumonia and who underwent BALF mNGS testing at the RICU of the First Affiliated Hospital of Henan University of Science and Technology from February 2022 to January 2024. Exclusion criteria included one patient under 18 years of age, 18 patients with multiple repeat detections of mNGS, 21 patients with incomplete clinical data, 10 patients failing to pass the quality control of mNGS, and 15 patients who died due to withdrawal of treatment. Ultimately, 236 patients were included in the study. Among these patients, 89 died within 28 days of hospitalization, while 147 survived, resulting in a 28-day mortality rate of 37.7%. The patient enrollment process is illustrated in **Figure 1**.

Comparisons of demographic and clinical characteristics between the survival and non-survival groups are summarized in **Table 1**. The study population had a mean age of 71.38 (SD 14.65) years, with 70.7% being male. Overall, 89 patients (37.7%) died during the 28-day follow-up. Non-survival patients had a higher prevalence of hypertension compared to survivors (53.9% vs 39.5%, *P* = 0.03). However, both survival and non-survival patients exhibited similar demographic characteristics (age, sex) and baseline comorbidities (such as diabetes, COPD, coronary artery disease, malignancy, etc.). Regarding laboratory test results, non-survival group had lower platelet counts and higher PCT levels compared to survival group. However, there were no significant differences in white blood cell count, lymphocyte count, neutrophil percentage, lymphocyte percentage, and CRP between the two groups (*P* > 0.05). In terms of admission scores, non-survivors had higher APACHE II and SOFA scores (*P* < 0.05), while there was no difference in CURB-65 score between the two groups (*P* > 0.05). Additionally, we compared the differences in length of hospital stay, ICU stay, duration of mechanical ventilation, and total hospitalization costs between the survival and non-survival groups. The results showed that these indicators were significantly higher in the non-survival group compared to the survival group (**Table 1**).

**Table 1:** Demographics and clinical characteristics of the study cohort.

Characteristics	Severe pneumonia (n=236)	Non-survival (n=89)	Survival (n=147)	*P* values
Age, years	71.38 ± 14.65	73.19 ± 13.30	70.29 ± 15.35	0.140
Male, n(%)	167 (70.7)	57 (64.0)	110 (74.8)	0.077
Comorbidities				
Hypertension, n(%)	106 (44.9)	48 (53.9)	58 (39.5)	0.030
DM, n(%)	78 (33.0)	35 (39.3)	78 (33.1)	0.111
COPD, n(%)	48 (20.3)	15 (16.9)	48 (20.3)	0.301
CAD, n(%)	81 (34.3)	37 (41.6)	44 (29.9)	0.068
Stroke, n(%)	75 (31.7)	31 (34.8)	44 (29.9)	0.443
Cancer, n(%)	48 (20.3)	16 (18.0)	32 (21.8)	0.483
Influenza, n(%)	22 (9.3)	9 (10.1)	13 (8.8)	0.745
COVID-19, n(%)	57 (24.1)	24 (27.0)	33 (22.4)	0.432
Laboratory detection				
WBC, x10^9/L	10.66 ± 5.89	10.48 ± 5.30	10.78 ± 6.26	0.712
NEU, %	83.68 ± 14.29	84.62 ± 14.76	83.08 ± 13.99	0.431
LYM, x10^9/L	0.82 ± 0.76	0.75 ± 0.72	0.87 ± 0.78	0.239
LYM%, %	10.18 ± 11.12	9.80 ± 12.65	10.43 ± 10.05	0.677
PLT, x10^12/L	182 (137,232)	175 (124.5,214)	187 (142.5,257)	0.026
PCT, ng/ml	0.30 (0.12,1.61)	0.38 (0.16, 2.23)	0.23 (0.11,0.91)	0.005
CRP, ng/ml	70.05 (30.23,110.22)	73.66 (35.95,120.40)	61.43 (22.54,104.55)	0.076
Disease severity assessment				
APACHEII score	25.69 ± 7.41	27.80 ± 7.20	24.35 ± 7.07	<0.001
SOFA score	5 (3,7)	6 (4,10)	4 (3, 7)	0.001
CURB-65 score	3.98 ± 0.87	4.08 ± 0.92	3.92 ± 0.83	0.169
Others				
LOS, days	20 (12,35)	13 (7, 20)	30 (16, 48)	<0.001
LOIS, days	11 (4,21)	9 (3, 14.5)	14 (5, 30)	<0.001
Duration of MV, hours	213.0 (48.8,483.8)	158.0 (64.5,321.0)	246.0 (48.0,692.0)	0.034
Costs, million(CNY)	8.12 (4.27,18.56)	7.44 (3.55,12.46)	9.43 (4.65, 26.33)	0.01

DM, diabetes mellitus; COPD, chronic obstructive pulmonary disease; CAD, coronary artery disease; COVID-19, coronavirus disease 2019; WBC, white blood cell count; NEU, neutrophilic granulocyte percent; LYM, lymphocyte count; LYM%, lymphocyte percentage; PLT, platelet count; PCT, procalcitonin; CRP, C-reactive protein; CURB-65 score, consists of five parameters, namely confusion, blood urea nitrogen (BUN) > 7 mmol/L, respiratory rate ≥ 30 breaths/minute, systolic blood pressure < 90 mmHg or diastolic blood pressure < 60 mmHg, and age ≥ 65 years. Each parameter met earns 1 point, with a total score ranging from 0 to 5 points. LOS: length of stay; LOIS, Length of ICU stay; MV, mechanical ventilation; CNY, China Yuan.

### 3.2 Microbial composition and diversity in survival and non-survival groups

The diversity and species composition were analyzed. The abundance level is based on the relative percentage of reads, and the analysis of alpha/beta diversity is based on the reads table. In this study, the microbial diversity of survival and non-survival group was analyzed (**Supplementary Figure S1A**). The results showed that there was no statistically significant difference between the two groups in alpha diversity (Chao1 and Shannon) (**Supplementary Figure S1C**). Similarly, the analysis of the beta diversity calculated with PCoA based on the Bray-Curtis metrics also showed no difference in the two groups (**Supplementary Figure S1D**). These results suggest that there is no significant difference in overall microbial diversity between survival and non-survival patients.

**Figure 2:**
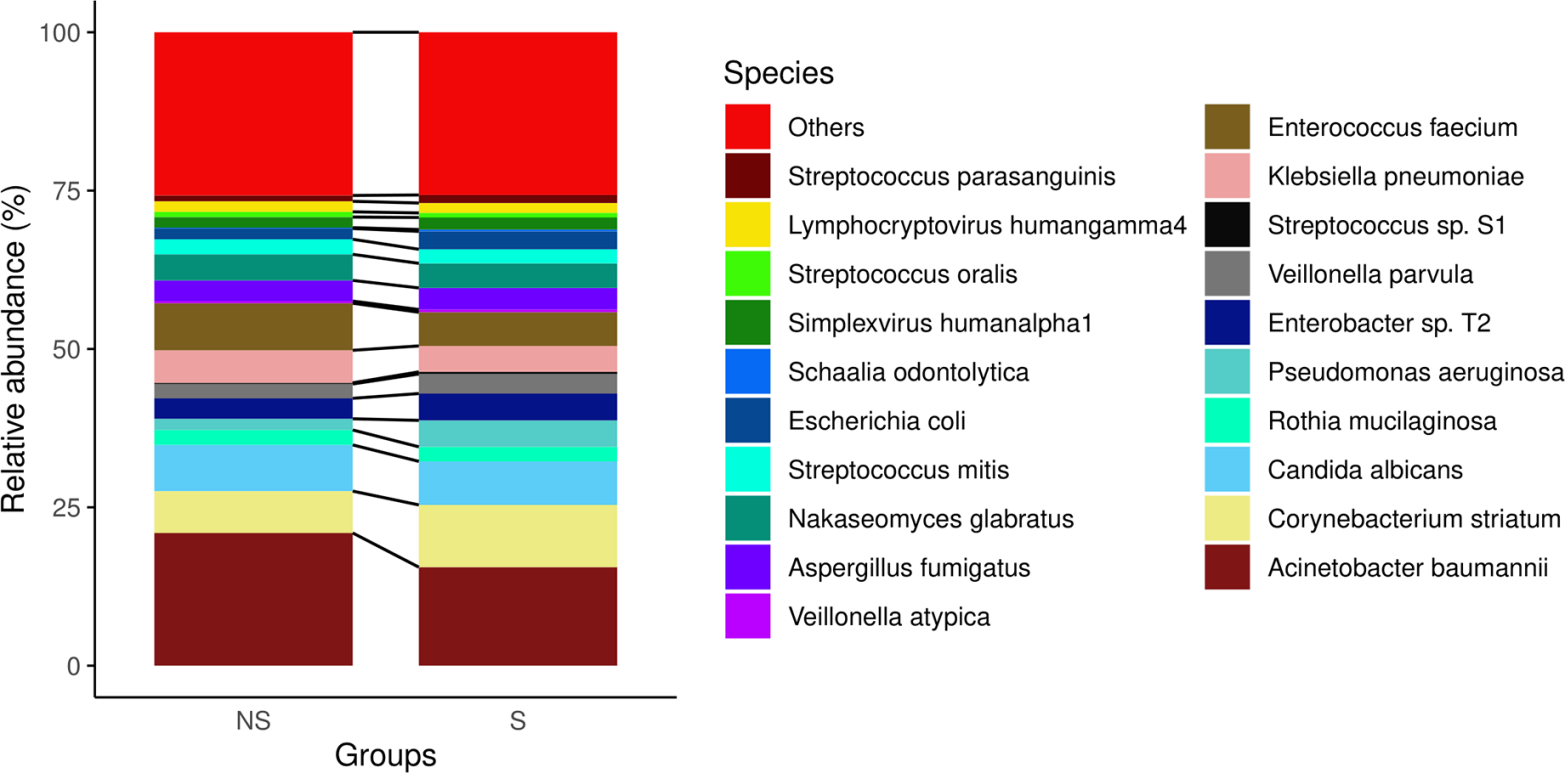
The composition of the pulmonary microbiome at the species level.

However, we observed some diversity patterns in both genera (**Figure 1**) and species level (**Figure 2**) between the two groups. Notably, the non-survival group exhibited a higher percentage of Acinetobacter baumannii, Klebsiella pneumoniae, and Enterococcus faecium. Conversely, the survival group showed a higher percentage of Corynebacterium striatum and Enterobacter. the non-survival group had a higher proportion of Acinetobacter baumannii, Klebsiella pneumoniae, and Enterococcus faecium, whereas the survival group displayed a higher prevalence of Corynebacterium striatum and Enterobacter (**Figure 2**). These findings imply persistent distinctions in microbial diversity between survivors and non-survivors.

### 3.3 Bacterial differences in the two groups

**Figure 3:**
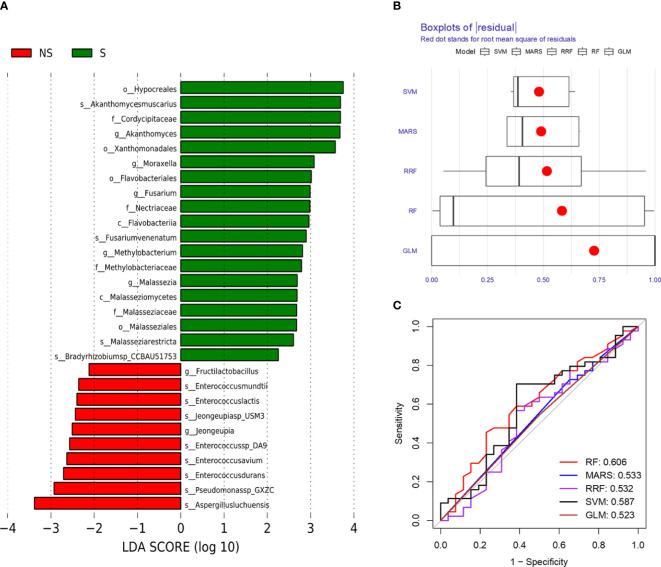
Bacterial biomarkers were identified by linear discriminant analysis effect size (LEfSe) and machine learning algorithm. **(A)** Bacterial histograms of unique biomarkers based on |LEfSe| >2. **(B)** Boxplots of residual distribution of each machine learning model. **(C)** ROC analysis of five machine learning models based on 5-fold cross-validation. The areas under the AUC were obtained for the five models.

We further analyzed the bacterial community structure associated with the non-survival and survival groups using LEfSe, an algorithm for high abundance biomarker discovery that uses linear discriminant analysis (LDA) to estimate the effect size of each taxon that differed between the two groups (**Figure 3**). A total of 29 distinct terms were identified. For the non-survival group, there were ten identified potential markers, mainly including Pseudomonassp, Jeongeupia and Enterococcusdurans. Hypocreales and Aspergillusluchuensishad the highest LDA scores, indicating a potential strong influence of microbial relative abundance in the survival group and non-survival group, respectively (**Figure 3A**).

### 3.4 Potential bacteria selected by machine learning model

**Figure 4:**
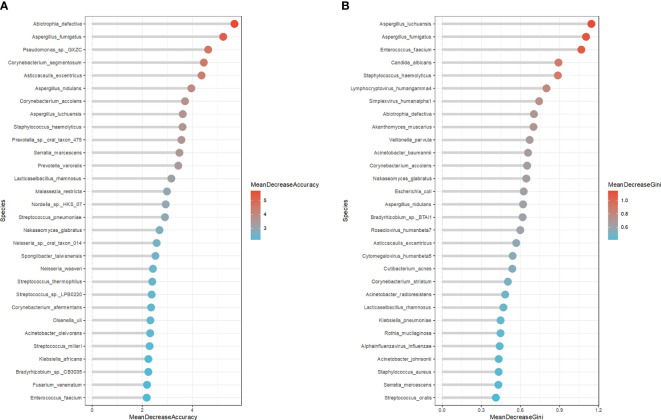
The potential microbe identified by RF model to predicate the short-term outcome of the severe pneumonia. **(A)** The top 30 potential pathogenic bacteria based on MeanDecreaseAccuracy analysis. **(B)** The top 30 potential pathogenic bacteria based on MeanDecreaseGini analysis.

To comprehensively describe the microbial characteristics between the non-survival group and the survival group, we initially applied five machine learning methods to determine the best-performing model. The residual distribution and ROC curve of each model were plotted. Among these models, the RF machine learning model exhibited the lower residuals and the highest area under the curve (AUC) in predicting the 28-day mortality of severe pneumonia (**Figures 3B, C**). Subsequently, RF was utilized to identify key pathogenic bacteria distinguishing the non-survival group from the survival group. Thirty potential pathogenic bacteria were screened by the random forest model, based on the analysis of MeanDecreaseAccuracy and MeanDecreaseGini. Through the assessment of these microorganisms’ pathogenic characteristics, we identified 16 pathogens,including Pseudomonas sp. GXZC, Corynebacterium segmentosum, Asticcacaulis excentricus, Klebsiella pneumoniae, Acinetobacter baumannii, Aspergillus fumigatus, Aspergillus nidulans, Streptococcus pneumoniae, Enterococcus faecium, Lymphocryptovirus humangamma4, Cytomegalovirus humanbeta5, Alphainfluenzavirus influenzae, Staphylococcus aureus, Enterococcus faecium, Prevotella veroralis and Malassezia restricta), which significantly contributed to predicting the short-term mortality of severe pneumonia patients (**Figure 4**).

### 3.5 Microbial interaction modes in the non-survival and survival groups

**Figure 5:**
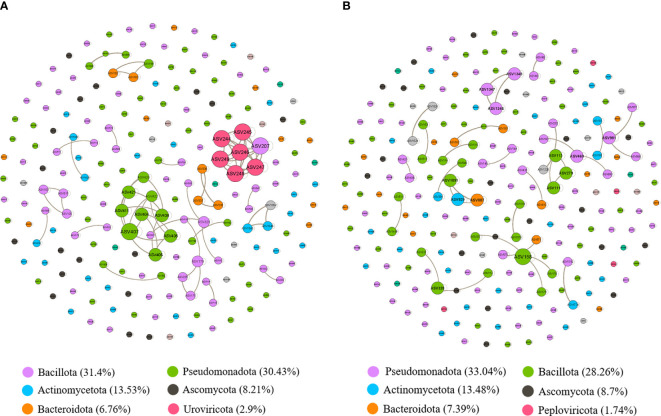
The correlation-based networks of abundant and frequent OTUs (relative abundance > 0.01%) for the two groups. **(A)** Network co-occurrence diagram between microbes in the non-survival patients. **(B)** Network co-occurrence diagram between microbes in the survival patients. The size of each node is proportional to the number of connections (i.e., degree), and the nodes are colored according to different phyla. Numbers inside parentheses following names of each phylum represent relative proportion of nodes belonging to the phylum. Grey edges indicate positive correlations.

We conducted further analysis by constructing a network co-occurrence map using Spearman correlation analysis, focusing on bacteria with relative abundances exceeding 0.01%. We considered correlations significant at a threshold of *P* < 0.05 and |r| > 0.8. In **Figure 5A**, the microbial network of the non-survival group comprised 207 nodes and 277 edges, while the survival group exhibited 230 nodes and 263 edges (**Figure 5B**). However, distinguishing between the complexity of the two groups was challenging. Subsequently, we calculated the average clustering coefficients (Bastian et al., 2009) (AvgCC) for both groups. The AvgCC values for the NS and S groups were 0.864 and 0.596, respectively, indicating greater complexity in the non-survival group compared to survival group. The top 6 phylum in the non-survival and survival groups were also showed in the bottom of **Figures 5A, B**, respectively. Then, we calculated the central genera bashed on the proportion of the connection number in each node (i.e., degree) at the genus level. In the non-survival group, the central genera in the network were Lactobacillus and Acinetobacter, with positive correlations within the respective species (**Supplementary Table S1**). Conversely, in the survival group, Streptococcus and Veillonella emerged as the central genera, exhibiting positive correlations within their respective sub-species (**Supplementary Table S2**). These findings imply that disparities in microbial interactions between non-survival and survival patients may contribute to differences in the short-term prognosis of severe pneumonia.

## 4 Discussion

The relationship between lung microbiota and the prognosis of severe pneumonia is a relatively understudied area. Despite the increasing attention to the role of the microbiome in respiratory diseases (Wang et al., 2016; Cuthbertson et al., 2020; Natalini et al., 2023), its correlation with the short-term prognosis of severe pneumonia remains unclear. Current research predominantly targets identifying pathogenic microorganisms to enhance antibiotic treatment for pneumonia (Xu et al., 2023). However, there is a relatively limited grasp of the association between microbiota and patient prognosis. In this study, we categorized patients into survival and non-survival groups based on whether they experienced death within 28 days. We investigated the differences in baseline characteristics between the two groups and particularly focused on examining the disparities in microbial composition. Additionally, leveraging machine learning strategies, we identified certain microbes that have the potential to distinguish or predict the short-term mortality risk of patients with severe pneumonia, providing new insights into severe pneumonia pathogenesis, prognosis, and the application of mNGS.

Severe pneumonia stands as one of the foremost causes of mortality worldwide, placing a significant burden on healthcare systems globally. The mortality rates among severe pneumonia patients range from 20% to 50%, underscoring the severity and complexity of this condition (Lee et al., 2020, Rodriguez et al., 2009, Valles et al., 2014; Walden et al., 2014). In our study, the observed mortality rate within 28 days of admission was 37.7%. However, it is essential to acknowledge that the actual mortality in severe pneumonia cases may be higher, as most severe pneumonia patients who did not undergo mNGS testing were excluded from our study. Additionally, various clinical scoring systems have been developed to predict mortality in critically ill patients, including those with severe pneumonia. The APACHE II and SOFA scores are well-established tools that have been validated (Raith et al., 2017; Wei et al., 2024) and widely recognized for prognostic prediction in critically ill patients (Ferreira et al., 2001; Izumida and Imamura, 2020; Tian et al., 2021). Consistent with previous findings, our study found that both APACHE II and SOFA scores were associated with mortality in severe pneumonia patients within 28 days of admission.

Furthermore, our results revealed no significant differences between the survival and non-survival groups in terms of gender, age, comorbidities (except hypertension), CURB-65 score, and most laboratory test results. However, among patients who died within 28 days, higher levels of PCT and PLT, shorter hospital stays, ICU stays, and mechanical ventilation durations, as well as higher hospitalization costs, were observed. These findings are consistent with previous studies. PCT and PLT could predict mortality in severe pneumonia patients (Mirsaeidi et al., 2010; Liu et al., 2016).

The primary objective of this study is to investigate the differences in lung microbiota between the non-survival and survival groups of severe pneumonia. Initially, we explored the alpha and beta diversity between the two groups, revealing no significant differences. Despite this apparent similarity, the lack of distinction between them may be attributed to sample size or sequencing biases, warranting further investigation. However, we did observe differences at the phylum level and species level. At the phylum level, Basidiomycota showed variance between the groups (**Supplementary Figure S2**). Although cases of Basidiomycota causing pneumonia are rare, there have been reported instances (Kim et al., 2022). At the species level, the non-survival group exhibited a higher percentage of Acinetobacter baumannii, Klebsiella pneumoniae, and Enterococcus faecium, which are commonly associated with drug resistance or multidrug resistance (De Oliveira et al., 2020), leading to substantial healthcare costs and adverse outcomes. It is estimated that approximately 541,000 deaths in Europe in 2019 were related to antibiotic resistance (European Antimicrobial Resistance Collaborators, 2022). Conversely, the survival group demonstrated a higher percentage of Corynebacterium striatum and Enterobacter. Corynebacterium striatum can also precipitate severe pneumonia or mortality, particularly in immunocompromised patients (Roig-Rico et al., 2011).

LEfSe analysis and machine learning methods serve as valuable tools for feature selection and biomarker screening. These approaches are instrumental in identifying key microbial signatures associated with different clinical outcomes in severe pneumonia patients. LEfSe analysis enables the detection of statistically significant differences in microbial abundance between groups, facilitating the identification of potential biomarkers. Our result showed that the non-survival group identified major potential pathogens, such as Pseudomonassp, Jeongeupia and Enterococcusdurans. The results regarding Enterococcusdurans replicated the findings of the diversity analysis between the two groups.

The machine learning strategy is a highlight of this study. Initially, we employed six machine learning models and randomly partitioned our mNGS data into training (70%) and validation (30%) sets. Subsequently, we utilized the residual distribution and ROC curve of each model to select the machine learning model with the lower residual distribution and highest AUC, identifying the random forest algorithm as the most suitable model for predicting the 28-day mortality risk of severe pneumonia patients. Subsequently, we systematically analyzed our data using the RF strategy. Based on the RF analysis of MeanDecreaseAccuracy and MeanDecreaseGini, and the subsequent assessment of these microorganisms’ pathogenic characteristics, we identified 16 pathogens, including Corynebacterium segmentosum, Asticcacaulis excentricus, Klebsiella pneumoniae, Acinetobacter baumannii, Enterococcus faecium, Lymphocryptovirus humangamma4, Cytomegalovirus humanbeta5, and Alpha influenza virus influenzae. In addition to partially overlapping with the previous results, these findings provided new evidence for predicting the short-term mortality of severe pneumonia, such as Cytomegalovirus humanbeta5 and Alpha influenza virus. Reactivation of cytomegalovirus can lead to serious adverse consequences in critically ill patients and immunocompromised individuals (Park et al., 2021; Liu et al., 2023). Cytomegalovirus infection-associated pneumonia has a high incidence rate (Coisel et al., 2012) and carries a higher mortality rate in immunodeficient patients (Huang and Tang, 2021; Lecuyer et al., 2022). Additionally, influenza, a potentially deadly infectious disease that has affected humans for centuries (Uyeki et al., 2022), is responsible for up to 650,000 annual deaths worldwide (Iuliano et al., 2018). Although influenza is usually self-limited, 5-10% of patients require ICU admission for additional supportive treatment (Beumer et al., 2019). In the ICU, influenza infections have a mortality rate of 20% (Sarda et al., 2019). Influenza infections are often followed by secondary bacterial pneumonia or complicated infections, known as a major cause of morbidity and mortality during influenza virus epidemics (Joseph et al., 2013; Sumitomo et al., 2021), which indicates the poor outcome of severe pneumonia combined with influenza.

Severe pneumonia represents not only single microbial infections but also perturbations in the entire lung microecosystem. Therefore, it is crucial to emphasize the lung’s microecosystem, which comprises diverse bacterial populations. Hence, alongside individual microbial infections, we should pay closer attention to the overall ecological changes within the lungs and the interrelations and interactions among various bacterial species. In this study, bacterial genera with a relative abundance greater than 0.01% were screened. Correlation analysis was performed for each genus, and the results were visualized using a network diagram. Our results showed that the microbial network of the non-survival group was more complex than the survival group. While a previous study suggested that the microbial network of mild pneumonia was more complex than that of severe pneumonia (Zhan et al., 2023), it’s worth noting that their study did not include mortality data, and the enrollment criteria of our study differed from theirs. The central genera in the non-survival group, Lactobacillus and Acinetobacter, are more likely to be core genera predicting 28-day mortality in severe pneumonia. Conversely, in the survival group, Streptococcus and Veillonella emerged as the central genera. Furthermore, both the central genera of the non-survival and survival groups were positively correlated with their respective species, with no correlation observed among other genera. This may be associated with sample size and sequencing biases. However, the central genera identified in the non-survival group showed certain consistency with the results of diversity analysis, LEfSe analysis, and machine learning strategies, indicating the credibility of the results.

With the widespread adoption of mNGS, accessing information about the lung microecosystem has become more convenient. However, mNGS is primarily utilized for diagnosing pathogens in lung infections, potentially overlooking crucial information about lung microecosystem dynamics. This study integrated lung microecosystem fluctuations with severe pneumonia prognosis, providing clinicians with a fresh perspective and new applications for mNGS technology.

Nevertheless, several limitations need to be acknowledged. Firstly, the relatively small sample size may limit the generalizability of the findings. Secondly, our study did not comprehensively analyze baseline comorbidities and other laboratory results alongside microbial data. Finally, this is a single-center study, which may restrict the generalizability to other healthcare settings with differing patient populations and treatment practices.

In summary, our study highlights the potential significance of lung microbiota composition in predicting the short-term prognosis of severe pneumonia patients. Through mNGS analysis, we identified distinct microbial profiles between these non-survival and survival groups. In the non-survival group, the presence of Acinetobacter baumannii, Klebsiella pneumoniae, Enterococcus faecium, and certain viruses may serve as potential predictors or markers for a worse outcome in patients with severe pneumonia. These findings underscore the potential role of the pulmonary microbiome in influencing the short-term prognosis of severe pneumonia. However, further research is needed to elucidate the underlying mechanisms and validate the clinical implications of these microbial differences.

## Data availability statement

The datasets presented in this study can be found in online repositories. The names of the repository/repositories and accession number(s) can be found below: https://db.cngb.org/search/project/ accession number: CNP0005426.

## Ethics statement

The studies involving humans were approved by The studies involving humans were approved by Research Ethics Committee of The First Affiliated Hospital of Henan University of Science and Technology. The studies were conducted in accordance with the local legislation and institutional requirements. The participants provided their written informed consent to participate in this study. Written informed consent was obtained from the individual(s) for the publication of any potentially identifiable images or data included in this article.

## Author contributions

S-SH: Formal analysis, Methodology, Writing – original draft, Software, Supervision, Visualization, Writing – review & editing. J-YQ: Investigation, Supervision, Validation, Visualization, Writing – review & editing. S-PL: Investigation, Methodology, Software, Writing – review & editing. Y-QM: Data curation, Project administration, Writing – review & editing. JH: Data curation, Formal analysis, Investigation, Writing – review & editing. L-NH: Data curation, Formal analysis, Investigation, Supervision, Writing – review & editing. L-LJ: Data curation, Formal analysis, Investigation, Resources, Writing – review & editing. CX: Data curation, Formal analysis, Investigation, Methodology, Software, Writing – review & editing. Y-MM: Conceptualization, Supervision, Validation, Writing – review & editing. Y-MZ: Conceptualization, Funding acquisition, Investigation, Project administration, Resources, Supervision, Validation, Writing – review & editing.

## Supplementary material

Supplementary Figure 1
**(A)** The heatmap of top 20 species in non-survival and survival groups. **(B)** Composition of pulmonary microorganisms at the generic level. **(C)** The Alpha diversity metrics (Shannon’s index and Chao1) between the two groups. **(D)** The beta diversity metric (PCoA) between the two groups.

Supplementary Figure 2 The difference in composition between the two groups at the phylum level.

## References

[B1] Antimicrobial Resistance Collaborators (2022). Global burden of bacterial antimicrobial resistance in 2019: a systematic analysis. Lancet 399, 629–655. doi: 10.1136/ebnurs-2022-103540 35065702 PMC8841637

[B2] Bastian M. Heymann S. Jacomy M. (2009). Gephi: an open source software for exploring and manipulating networks. doi: 10.1609/icwsm.v3i1.13937

[B3] Beumer M. C. Koch R. M. van Beuningen D. OudeLashof A. M. van de Veerdonk F. L. Kolwijck E.. (2019). Influenza virus and factors that are associated with ICU admission, pulmonary co-infections and ICU mortality. J. Crit. Care 50, 59–65. doi: 10.1016/j.jcrc.2018.11.013 30481669 PMC7125534

[B4] Budden K. F. Shukla S. D. Rehman S. F. Bowerman K. L. Keely S. Hugenholtz P.. (2019). Functional effects of the microbiota in chronic respiratory disease. Lancet Respir. Med. 7, 907–920. doi: 10.1016/S2213-2600(18)30510-1 30975495

[B5] Chen J. Zhang R. Liu L. Qi T. Wang Z. Song W.. (2021). Clinical usefulness of metagenomic next-generation sequencing for the diagnosis of central nervous system infection in people living with HIV. Int. J. Infect. Dis. 107, 139–144. doi: 10.1016/j.ijid.2021.04.057 33892189

[B6] Chiu C. Y. Miller S. A. (2019). Clinical metagenomics. Nat. Rev. Genet. 20, 341–355. doi: 10.1038/s41576-019-0113-7 30918369 PMC6858796

[B7] Cilloniz C. Torres A. Niederman M. S. (2021). Management of pneumonia in critically ill patients. BMJ 375, e065871. doi: 10.1136/bmj-2021-065871 34872910

[B8] Coisel Y. Bousbia S. Forel J. M. Hraiech S. Lascola B. Roch A.. (2012). Cytomegalovirus and herpes simplex virus effect on the prognosis of mechanically ventilated patients suspected to have ventilator-associated pneumonia. PloS One 7, e51340. doi: 10.1371/journal.pone.0051340 23236477 PMC3517464

[B9] Cuthbertson L. Walker A. W. Oliver A. E. Rogers G. B. Rivett D. W. Hampton T. H.. (2020). Lung function and microbiota diversity in cystic fibrosis. Microbiome 8, 45. doi: 10.1186/s40168-020-00810-3 32238195 PMC7114784

[B10] De Oliveira D. M. P. Forde B. M. Kidd T. J. Harris P. N. A. Schembri M. A. Beatson S. A.. (2020). Antimicrobial resistance in ESKAPE pathogens. Clin. Microbiol. Rev. 17, 33. doi: 10.1128/CMR.00181-19 PMC7227449 32404435

[B11] Diseases G. B. D. Injuries C. (2020). Global burden of 369 diseases and injuries in 204 countries and territories 1990-2019: a systematic analysis for the Global Burden of Disease Study 2019. Lancet 396, 1204–1222. doi: 10.1016/S0140-6736(20)30925-9 33069326 PMC7567026

[B12] European Antimicrobial Resistance Collaborators (2022). The burden of bacterial antimicrobial resistance in the WHO European region in 2019: a cross-country systematic analysis. Lancet Public Health 7, e897–e913. doi: 10.1016/S2468-2667(22)00225-0 36244350 PMC9630253

[B13] Ferreira F. L. Bota D. P. Bross A. Melot C. Vincent J. L. (2001). Serial evaluation of the SOFA score to predict outcome in critically ill patients. JAMA 286, 1754–1758. doi: 10.1001/jama.286.14.1754 11594901

[B14] Huang W. J. Tang X. X. (2021). Virus infection induced pulmonary fibrosis. J. Transl. Med. 19, 496. doi: 10.1186/s12967-021-03159-9 34876129 PMC8649310

[B15] Hufnagl K. Pali-Scholl I. Roth-Walter F. Jensen-Jarolim E. (2020). Dysbiosis of the gut and lung microbiome has a role in asthma. Semin. Immunopathol. 42, 75–93. doi: 10.1007/s00281-019-00775-y 32072252 PMC7066092

[B16] Iuliano A. D. Roguski K. M. Chang H. H. Muscatello D. J. Palekar R. Tempia S.. (2018). Estimates of global seasonal influenza-associated respiratory mortality: a modelling study. Lancet 391, 1285–1300. doi: 10.1016/S0140-6736(17)33293-2 29248255 PMC5935243

[B17] Izumida T. Imamura T. (2020). The prognosis of critically ill patients with invasive group A streptococcus infection. Crit. Care 24, 437. doi: 10.1186/s13054-020-03167-z 32665008 PMC7362403

[B18] Jaafari A. Janizadeh S. Abdo H. G. Mafi-Gholami D. Adeli B. (2022). Understanding land degradation induced by gully erosion from the perspective of different geoenvironmental factors. J. Environ. Manage 315, 115181. doi: 10.1016/j.jenvman.2022.115181 35500480

[B19] Jain S. Self W. H. Wunderink R. G. Fakhran S. Balk R. Bramley A. M.. (2015). Community-acquired pneumonia requiring hospitalization among U.S. Adults. N Engl. J. Med. 373, 415–427. doi: 10.1056/NEJMoa1500245 26172429 PMC4728150

[B20] Jin X. Li J. Shao M. Lv X. Ji N. Zhu Y.. (2022). Improving suspected pulmonary infection diagnosis by bronchoalveolar lavage fluid metagenomic next-generation sequencing: a multicenter retrospective study. Microbiol. Spectr. 10, e0247321. doi: 10.1128/spectrum.02473-21 35943274 PMC9431624

[B21] Joseph C. Togawa Y. Shindo N. (2013). Bacterial and viral infections associated with influenza. Influenza Other Respir. Viruses 7 Suppl 2, 105–113. doi: 10.1111/irv.12089 24034494 PMC5909385

[B22] Kim H. Yi Y. Cho S. Y. Lee D. G. Chun H. S. Park C.. (2022). Pneumonia due to Schizophyllum commune in a Patient with Acute Myeloid Leukemia: Case Report and Literature Review. Infect. Chemother. 54, 195–201. doi: 10.3947/ic.2020.0068 33124214 PMC8987182

[B23] Langmead B. Wilks C. Antonescu V. Charles R. (2019). Scaling read aligners to hundreds of threads on general-purpose processors. Bioinformatics 35, 421–432. doi: 10.1093/bioinformatics/bty648 30020410 PMC6361242

[B24] Lecuyer R. Issa N. Tessoulin B. Lavergne R. A. Morio F. Gabriel F.. (2022). Epidemiology and Clinical Impact of Respiratory Coinfections at Diagnosis of Pneumocystis jirovecii Pneumonia. J. Infect. Dis. 225, 868–880. doi: 10.1093/infdis/jiab460 34604908

[B25] Lee H. W. Ji E. Ahn S. Yang H. J. Yoon S. Y. Park T. Y.. (2020). A population-based observational study of patients with pulmonary disorders in intensive care unit. Korean J. Intern. Med. 35, 1411–1423. doi: 10.3904/kjim.2018.449 31752478 PMC7652646

[B26] Li P. Luo H. Ji B. Nielsen J. (2022). Machine learning for data integration in human gut microbiome. Microb. Cell Fact 21, 241. doi: 10.1186/s12934-022-01973-4 36419034 PMC9685977

[B27] Li Y. Sun B. Tang X. Liu Y. L. He H. Y. Li X. Y.. (2020). Application of metagenomic next-generation sequencing for bronchoalveolar lavage diagnostics in critically ill patients. Eur. J. Clin. Microbiol. Infect. Dis. 39, 369–374. doi: 10.1007/s10096-019-03734-5 31813078 PMC7102353

[B28] Liu D. Su L. X. Guan W. Xiao K. Xie L. X. (2016). Prognostic value of procalcitonin in pneumonia: A systematic review and meta-analysis. Respirology 21, 280–288. doi: 10.1111/resp.12704 26662169 PMC4738441

[B29] Liu L. Oza S. Hogan D. Chu Y. Perin J. Zhu J.. (2016). Global, regional, and national causes of under-5 mortality in 2000-15: an updated systematic analysis with implications for the Sustainable Development Goals. Lancet 388, 3027–3035. doi: 10.1016/S0140-6736(16)31593-8 27839855 PMC5161777

[B30] Liu Y. Wen Z. Fang Y. Wang T. Wu F. Zhang H.. (2023). Herpesvirus reactivation in respiratory tract is associated with increased mortality of severe pneumonia patients and their respiratory microbiome dysbiosis. Front. Cell Infect. Microbiol. 13, 1294142. doi: 10.3389/fcimb.2023.1294142 38188628 PMC10771827

[B31] Mandell L. A. Wunderink R. G. Anzueto A. Bartlett J. G. Campbell G. D. Dean N. C.. (2007). Infectious Diseases Society of America/American Thoracic Society consensus guidelines on the management of community-acquired pneumonia in adults. Clin. Infect. Dis. 44 Suppl 2, S27–S72. doi: 10.1086/511159 17278083 PMC7107997

[B32] Meng L. N. Li G. Yuan H. X. Feng X. C. Liu F. Zhang S. L. (2023). Utility of metagenomics next-generation sequencing in the diagnosis and treatment of severe infectious diseases in the intensive care unit. Technol. Health Care 31, 1887–1899. doi: 10.3233/THC-220856 37302051

[B33] Metlay J. P. Waterer G. W. Long A. C. Anzueto A. Brozek J. Crothers K.. (2019). Diagnosis and treatment of adults with community-acquired pneumonia. An official clinical practice guideline of the american thoracic society and infectious diseases society of America. Am. J. Respir. Crit. Care Med. 200, e45–e67. doi: 10.1164/rccm.201908-1581ST 31573350 PMC6812437

[B34] Mirsaeidi M. Peyrani P. Aliberti S. Filardo G. Bordon J. Blasi F.. (2010). Thrombocytopenia and thrombocytosis at time of hospitalization predict mortality in patients with community-acquired pneumonia. Chest 137, 416–420. doi: 10.1378/chest.09-0998 19837825

[B35] Montassier E. Kitsios G. D. Radder J. E. Le Bastard Q. Kelly B. J. Panzer A.. (2023). Robust airway microbiome signatures in acute respiratory failure and hospital-acquired pneumonia. Nat. Med. 29, 2793–2804. doi: 10.1038/s41591-023-02617-9 37957375

[B36] Natalini J. G. Singh S. Segal L. N. (2023). The dynamic lung microbiome in health and disease. Nat. Rev. Microbiol. 21, 222–235. doi: 10.1038/s41579-022-00821-x 36385637 PMC9668228

[B37] Park G. E. Ki H. K. Ko J. H. (2021). Impact of antiviral treatment on long-term prognosis in non-immunocompromised patients with CMV reactivation. BMC Infect. Dis. 21, 414. doi: 10.1186/s12879-021-06098-4 33947335 PMC8094573

[B38] Peng J. M. Du B. Qin H. Y. Wang Q. Shi Y. (2021). Metagenomic next-generation sequencing for the diagnosis of suspected pneumonia in immunocompromised patients. J. Infect. 82, 22–27. doi: 10.1016/j.jinf.2021.01.029 33609588

[B39] Prina E. Ranzani O. T. Torres A. (2015). Community-acquired pneumonia. Lancet 386, 1097–1108. doi: 10.1016/S0140-6736(15)60733-4 26277247 PMC7173092

[B40] Raith E. P. Udy A. A. Bailey M. McGloughlin S. MacIsaac C. Bellomo R.. (2017). Prognostic accuracy of the SOFA score, SIRS criteria, and qSOFA score for in-hospital mortality among adults with suspected infection admitted to the intensive care unit. JAMA 317, 290–300. doi: 10.1001/jama.2016.20328 28114553

[B41] Rodriguez A. Lisboa T. Blot S. Martin-Loeches I. Sole-Violan J. De Mendoza D.. (2009). Mortality in ICU patients with bacterial community-acquired pneumonia: when antibiotics are not enough. Intensive Care Med. 35, 430–438. doi: 10.1007/s00134-008-1363-6 19066850

[B42] Roig-Rico P. Safont-Gaso P. Marin-Tordera D. Ortiz-De la Tabla V. (2011). [Corynebacterium striatum pneumonia in an HIV patient]. Enferm Infecc Microbiol. Clin. 29, 402. doi: 10.1016/j.eimc.2011.02.005 21477896

[B43] Sarda C. Palma P. Rello J. (2019). Severe influenza: overview in critically ill patients. Curr. Opin. Crit. Care 25, 449–457. doi: 10.1097/MCC.0000000000000638 31313681

[B44] Segata N. Izard J. Waldron L. Gevers D. Miropolsky L. Garrett W. S.. (2011). Metagenomic biomarker discovery and explanation. Genome Biol. 12, R60. doi: 10.1186/gb-2011-12-6-r60 21702898 PMC3218848

[B45] Shi C. L. Han P. Tang P. J. Chen M. M. Ye Z. J. Wu M. Y.. (2020). Clinical metagenomic sequencing for diagnosis of pulmonary tuberculosis. J. Infect. 81, 567–574. doi: 10.1016/j.jinf.2020.08.004 32768450

[B46] Song J. H. Huh K. Chung D. R. (2016). Community-acquired pneumonia in the Asia-Pacific region. Semin. Respir. Crit. Care Med. 37, 839–854. doi: 10.1055/s-0036-1592075 27960208 PMC7171710

[B47] Spindler C. Ortqvist A. (2006). Prognostic score systems and community-acquired bacteraemic pneumococcal pneumonia. Eur. Respir. J. 28, 816–823. doi: 10.1183/09031936.06.00144605 16737983

[B48] Sumitomo T. Nakata M. Nagase S. Takahara Y. Honda-Ogawa M. Mori Y.. (2021). GP96 drives exacerbation of secondary bacterial pneumonia following influenza A virus infection. mBio 12, e0326920. doi: 10.1128/mBio.03269-20 34061598 PMC8262878

[B49] Tian Y. Yao Y. Zhou J. Diao X. Chen H. Cai K.. (2021). Dynamic APACHE II score to predict the outcome of intensive care unit patients. Front. Med. (Lausanne) 8, 744907. doi: 10.21203/rs.3.rs-642050/v1 35155461 PMC8826444

[B50] Uyeki T. M. Hui D. S. Zambon M. Wentworth D. E. Monto A. S. (2022). Influenza. Lancet 400, 693–706. doi: 10.1016/S0140-6736(22)00982-5 36030813 PMC9411419

[B51] Valles J. Martin-Loeches I. Torres A. Diaz E. Seijas I. Lopez M. J.. (2014). Epidemiology, antibiotic therapy and clinical outcomes of healthcare-associated pneumonia in critically ill patients: a Spanish cohort study. Intensive Care Med. 40, 572–581. doi: 10.1007/s00134-014-3239-2 24638939 PMC7094988

[B52] Ventola C. L. (2015). The antibiotic resistance crisis: part 1: causes and threats. P T 40, 277–283. 25859123 PMC4378521

[B53] Walden A. P. Clarke G. M. McKechnie S. Hutton P. Gordon A. C. Rello J.. (2014). Patients with community acquired pneumonia admitted to European intensive care units: an epidemiological survey of the GenOSept cohort. Crit. Care 18, R58. doi: 10.1186/cc13812 24690444 PMC4056764

[B54] Wang Z. Bafadhel M. Haldar K. Spivak A. Mayhew D. Miller B. E.. (2016). Lung microbiome dynamics in COPD exacerbations. Eur. Respir. J. 47, 1082–1092. doi: 10.1183/13993003.01406-2015 26917613

[B55] Wei Z. Zhao L. Yan J. Wang X. Li Q. Ji Y.. (2024). Dynamic monitoring of neutrophil/lymphocyte ratio, APACHE II score, and SOFA score predict prognosis and drug resistance in patients with Acinetobacter baumannii-calcoaceticus complex bloodstream infection: a single-center retrospective study. Front. Microbiol. 15, 1296059. doi: 10.3389/fmicb.2024.1296059 38322313 PMC10844563

[B56] Wood D. E. Lu J. Langmead B. (2019). Improved metagenomic analysis with Kraken 2. Genome Biol. 20, 257. doi: 10.1186/s13059-019-1891-0 31779668 PMC6883579

[B57] Xie G. Zhao B. Wang X. Bao L. Xu Y. Ren X.. (2021). Exploring the clinical utility of metagenomic next-generation sequencing in the diagnosis of pulmonary infection. Infect. Dis. Ther. 10, 1419–1435. doi: 10.1007/s40121-021-00476-w 34117999 PMC8322361

[B58] Xu J. Zhou P. Liu J. Zhao L. Fu H. Han Q.. (2023). Utilizing metagenomic next-generation sequencing (mNGS) for rapid pathogen identification and to inform clinical decision-making: results from a large real-world cohort. Infect. Dis. Ther. 12, 1175–1187. doi: 10.1007/s40121-023-00790-5 36988865 PMC10147866

[B59] Yu W. Li S. Ye T. Xu R. Song J. Guo Y. (2022). Deep ensemble machine learning framework for the estimation of PM2.5 concentrations. Environ. Health Perspect. 130, 37004. doi: 10.1289/EHP9752 35254864 PMC8901043

[B60] Zhan D. Li D. Yuan K. Sun Y. He L. Zhong J.. (2023). Characteristics of the pulmonary microbiota in patients with mild and severe pulmonary infection. Front. Cell Infect. Microbiol. 13, 1227581. doi: 10.3389/fcimb.2023.1227581 37900322 PMC10602873

