# Supplementary figures and images for: Microbial signatures predictive of short-term prognosis in severe pneumonia
Source: Front Cell Infect Microbiol. 2024 Aug 2;14:1397717. doi: 10.3389/fcimb.2024.1397717 (PMC11327560; doi:10.3389/fcimb.2024.1397717)

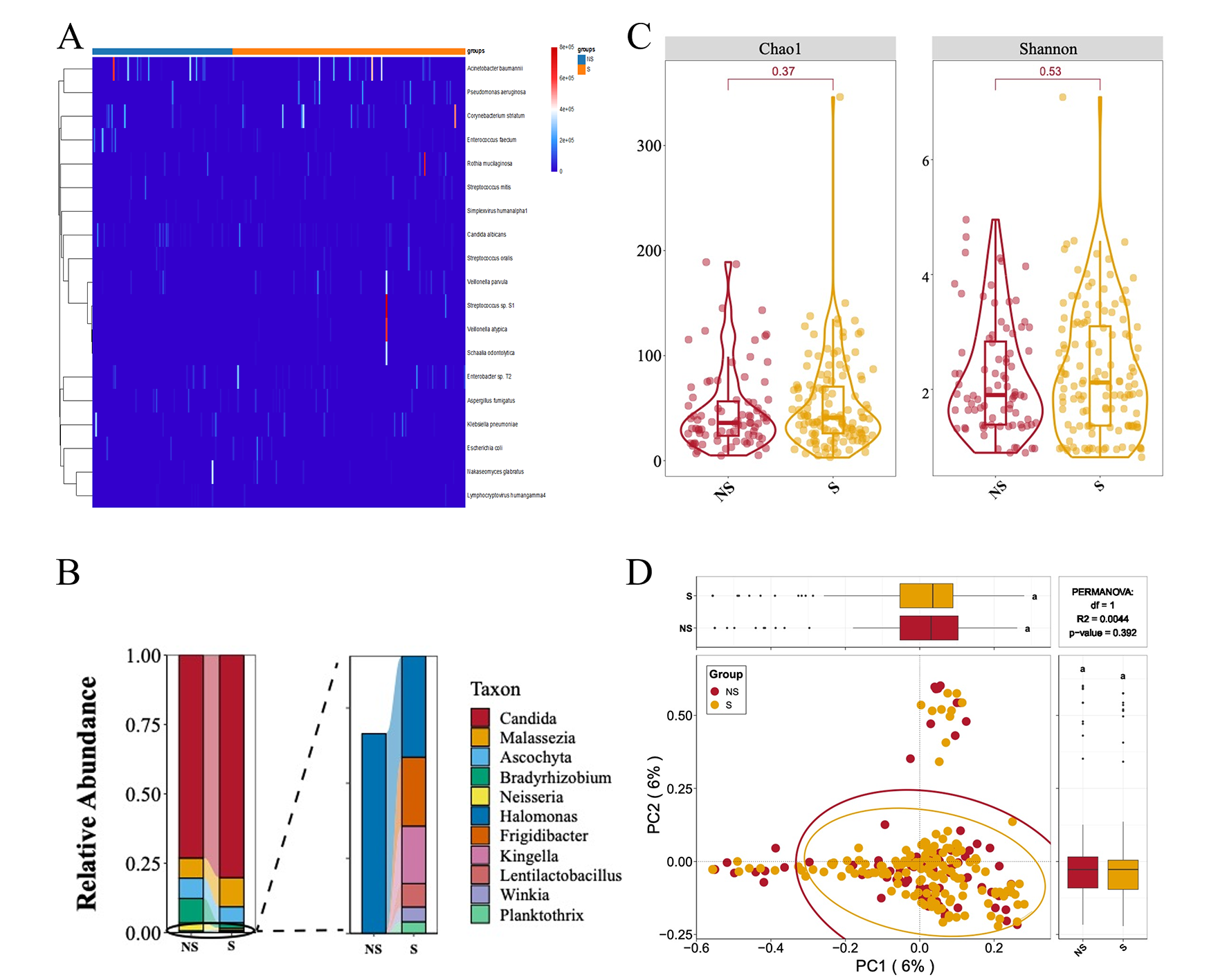

Supplement: Supplementary Figure 1 — (A) The heatmap of top 20 species in non-survival and survival groups. (B) Composition of pulmonary microorganisms at the generic level. (C) The Alpha diversity metrics (Shannon’s index and Chao1) between the two groups. (D) The beta diversity metric (PCoA) between the two groups. [file Image_1.tif]

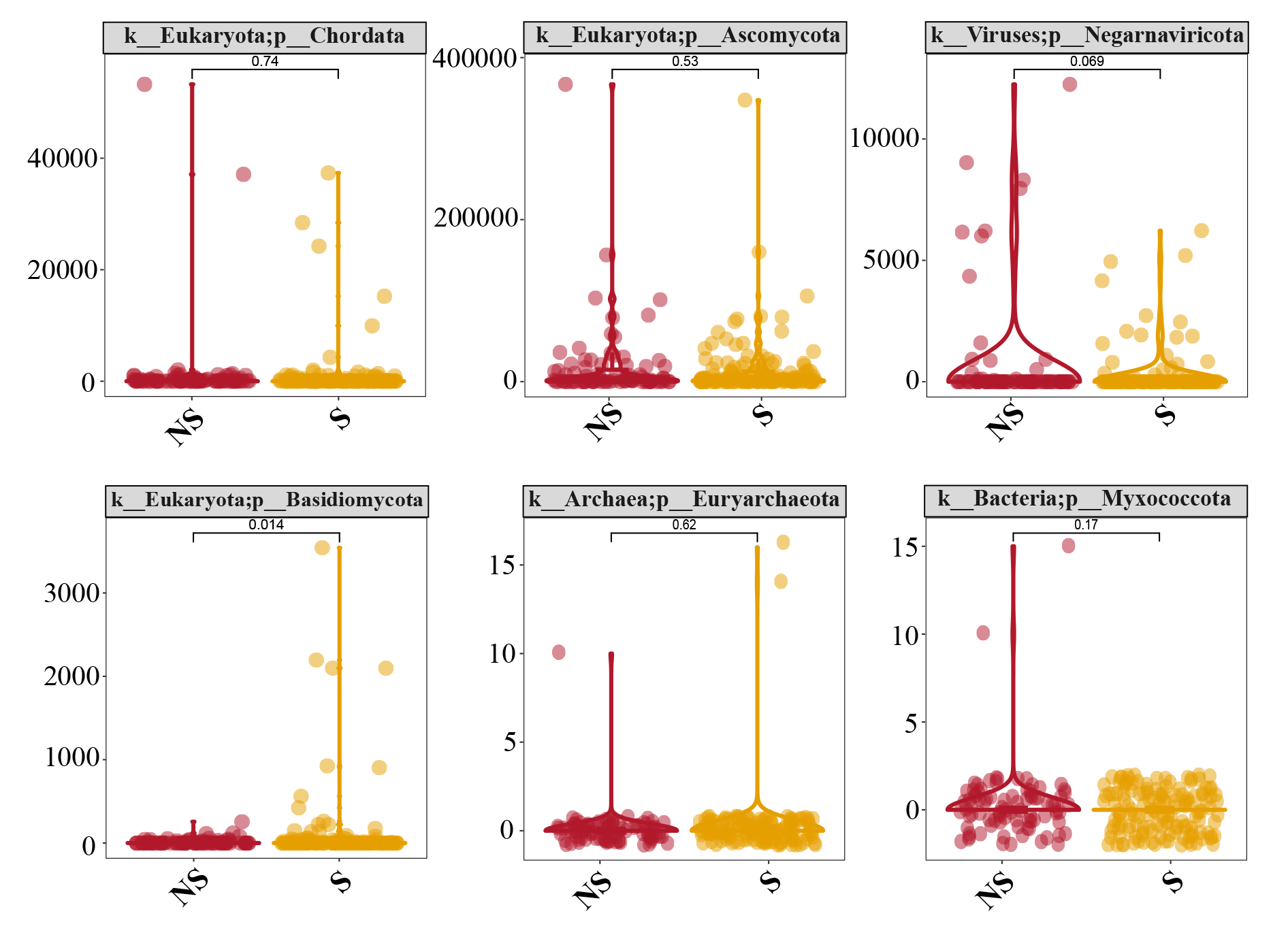

Supplement: Supplementary Figure 2 — The difference in composition between the two groups at the phylum level. [file Image_2.tif]
